# Use of Belladonna in Arresting Secretions of Milk in Swollen and Painful Breasts

**Published:** 1857-05

**Authors:** 


*Dr. S. Willey reports the following Use of Belladonna in arresting secretions of Milk in swollen and painful Breasts:*

St. Paul, January 4th, 1857.

Editors Medical Observer--Upon reading Dr. Goolden's article in my number of the *London Lancet* for October (not August,) I resolved to make early trial of the remedy, and five cases have been furnished me of tumefied and painful breasts, not differing in any essential feature from those reported By Dr. G., in which the free use of extract of belladonna to the areolà has acted like a specific in every instance. Once only did assistance seem required—citrate of magnesia having been given where protracted constipation obtained. Notes of the cases were kept without the remotest idea of publication, until I saw the article copied into the *Observer* for December. I hand you two extracts from my note book.

Mrs. B. aged 18, child aged 11 months, commenced menstruating regularly five months after birth of child, catamenia not occurring on 12th Nov. when expected, together with the appearance of an obstinate diarrhea in the child, induced weaning, which was soon followed by distended and painful breasts. Ordered that the breasts should be well drawn and an ointment composed of ext. belladonna and simple cerate, two-thirds of the former to one of the latter to be rubbed upon the nipples and areola every two hours. Three hours subsequently, found breasts flaccid and free from pain, much to the expressed wonder of the lady, and the concealed surprise of myself. No re-filling.

Mrs. P. aged 21, having lost second child aged seven months, suddenly suffered with red, turgid and throbbing breasts, pain much aggravated by riding to the cemetery, two miles distant, temperature 12° F. below zero. The application of belladonna *to the entire breasts* was made in this case and complete relief was obtained in six hours. Extreme dilatation of the pupils with strabismus, was produced, which passed away soon after the administration of paragoric in hot brandy sling. This was the only case where other than the nipples and areola were anointed.—*Cin. Med. Obs.*

